# Modelling Across Multiple Scales to Design Biopolymer Membranes for Sustainable Gas Separations: 2-Multiscale Approach

**DOI:** 10.3390/polym16192776

**Published:** 2024-09-30

**Authors:** Kseniya Papchenko, Eleonora Ricci, Maria Grazia De Angelis

**Affiliations:** 1 Institute for Materials and Processes, School of Engineering, University of Edinburgh, Sanderson Building, Robert Stevenson Road, Edinburgh EH9 3FB, UK; kpapchen@ed.ac.uk (K.P.); ericci@ed.ac.uk (E.R.); 2 Department of Civil, Chemical Environmental and Materials Engineering, DICAM, University of Bologna, Via Terracini 28, 40131 Bologna, Italy

**Keywords:** gas separation, biopolymers, multiscale modelling

## Abstract

The majority of materials used for membrane-based separation of gas mixtures are non-renewable and non-biodegradable, and the assessment of alternative bio-based polymers requires expensive and time-consuming experimental campaigns. This effort can be reduced by adopting suitable modelling approaches. In this series of works, we propose various modelling approaches to assess the CO_2_/CH_4_ separation performance of eight different copolymers of 3-hydroxybutyrate and 3-hydroxyvalerate (PHBV) using a limited amount of experimental data for model calibration. In part 1, we adopted a fully atomistic approach based on Molecular Dynamics (MD), while, in this work, we propose a multiscale methodology where a molecular description of the polymers is bridged to a macroscopic prediction of its gas sorption behaviour. PHBV structures were simulated using MD to obtain pressure–volume–temperature data, which were used to parametrise the Sanchez–Lacombe Equation of State. This, in turn, allows for the evaluation of the CO_2_ and CH_4_ solubility in the copolymers at various pressures and compositions with little computational effort, enabling the estimate of the sorption-based selectivity. The gas separation performance obtained with this multiscale technique was compared to results obtained with a fully atomistic model and experimental data. The solubility–selectivity for the CO_2_/CH_4_ mixture is in reasonable agreement between the two models and the experimental data. The multiscale method presented is a time-efficient alternative to fully atomistic methods and detailed experimental campaigns and can accelerate the introduction of renewable materials in different applications.

## 1. Introduction

The introduction of biopolymers in the membrane field is of increasing interest for industrially relevant applications such as gas separations [1,2,3,4]. Polymer-based materials with low environmental impact and minor disposal concerns would contribute to the overall sustainability of membrane processes, favoured by high energy efficiency and intrinsic environmentally responsible design [5,6]. Biopolymers can offer properties that are comparable to fossil-based counterparts in terms of material performance required for the specific application; however, the commercialisation of these as membrane materials requires the development of efficient strategies to find an optimal formulation with good mechanical, thermal, and transport properties [7]. PHAs are linear semicrystalline polyesters synthesised naturally by several microorganisms as an energy reserve, and they present thermo-mechanical properties similar to their synthetic counterparts [8]. Poly(3–hydroxybutyrate) (PHB), poly(3–hydroxyvalerate) (PHV), and their copolymers are currently the most studied and produced bio-polyesters, as the potential application range of such materials appears to be quite broad [9,10,11,12].

Given the high number of possible biopolymer candidates, systematic experimental studies are expensive and time-consuming. Alongside the experimental characterisation, the computational description of polymer-based systems is of great importance for the fundamental understanding of the physical mechanisms involved, as well as for practical design purposes, aiming at the development of novel membranes and the optimisation of the process operating conditions. Computational modelling can be an extremely useful tool for the characterisation of novel materials, as it enables the evaluation of the performance of materials and conditions not yet accessed experimentally and predicts structure–property relationships in a fast and reliable manner. In particular, several techniques have been proposed for modelling sorption and transport properties in polymeric materials across different scales [13,14]. Molecular Dynamics (MD) simulations have been proven efficient in predicting a range of polymer properties, including energetic parameters, such as cohesive energy density and penetrant solubility coefficients; kinetic parameters, such as penetrant diffusion coefficients and polymer segmental dynamics; structural parameters, such as static structure factor and radius of gyration; thermo-mechanical properties, such as Young’s modulus and glass transition temperature [15,16,17,18]. Macroscopic models, such as Equations of State (EoS), are known for their high predictive power and complete representation of the polymer–fluid mixture both in equilibrium and non-equilibrium conditions [19,20,21,22]. The polymeric system can be described in a multiscale manner if MD simulations are used to generate polymer data useful to parametrise EoS, and such an approach has already been successfully applied to conventional polymers like Polystyrene, Matrimid^®^, Ultem^®^, and Kapton^®^, allowing us to describe and predict gas solubility behaviour in these systems [18,23,24].

In part 1 of this series of papers, we used a fully atomistic description to assess the transport properties in polymers of the Poly(hydroxyalkanoate) (PHAs) family [25], focusing on the CO_2_/CH_4_ separation. In particular, we used the molecular approach called the Widom insertion method [26] to predict CO_2_ and CH_4_ solubility coefficients in the whole range of Poly(3–hydroxybutyrate–co–3–hydroxyvalerate) (PHBV) copolymer compositions after a rigorous validation of the model on data available in the literature [27,28,29,30,31,32]. The advantage of using a molecular approach to compute gas solubility lies in its entirely predictive character and, thus, its applicability to cases for which experimental data are not available. On a negative note, MD simulations of polymers are very computationally intensive and need to be repeated for each gas or copolymer composition considered, which somehow limits the amount of data collectable in reasonable timeframes.

In this work, we propose a multiscale framework that, thanks to its higher computational efficiency with respect to MD, allows us to increase the productivity and quality of the simulations by extending the prediction performed in part 1 to a larger set of PHBV copolymers, and by representing additional details of the process, such as the effect of gas–gas interactions during multicomponent sorption.

The multiscale approach makes use of the atomistic model created in part 1 [25] to generate pressure–volume–temperature (PVT) data of the copolymers that are fed into the macroscopic Sanchez–Lacombe (SL) EoS [19,20], which is fitted on them to obtain three characteristic parameters for each copolymer. The predictions of gas solubility from the SL EoS are then compared with experimental and MD data previously obtained [25] and are used to expand the gas separation predictions under mixed-gas conditions relevant to membrane separation.

This is the first time that such a multiscale approach has been applied to biopolymers, which are characterised by a chronic lack of sorption and transport data needed for the design of membrane processes. Moreover, the gas sorption properties of PHAs were never modelled before using an EoS, to the best of the authors’ knowledge. Therefore, we believe that the approach presented and demonstrated here for the case of PHAs will unlock a new method to screen the applicability of novel biopolymers to membrane processes and other applications, such as packaging.

## 2. Materials and Methods

### 2.1. Materials

**Figure 1:**
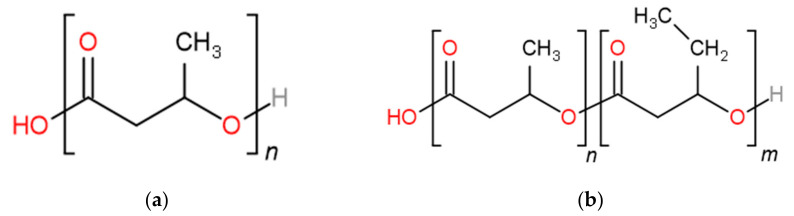
Chemical structure of (**a**) Poly(3–hydroxybutyrate) (PHB) and (**b**) copolymer of Poly(3–hydroxybutyrate) and Poly(3–hydroxyvalerate) (PHBV).

Polymers of the PHA family represent an attractive alternative to traditional petroleum-based materials, as their origin is fully renewable, and biodegradability can be achieved in many environments [8,33,34]. PHAs are linear semicrystalline biopolyesters able to offer properties comparable to those of commercially relevant polymers, with potential applicability in fields ranging from biomedical applications to food packaging to additive manufacturing [8,9,10,11,12,25,34,35]. PHBV copolymers present a relatively high crystallinity degree of 40 ÷ 70% and are generally above their $T_{g}$ at ambient conditions, with melting occurring between 100 and 180 °C and thermal degradation between 270 and 320 °C [12,36,37]. In the present work, PHBV copolymers with 0 to 100% mol% of 3–hydroxyvalerate (HV) units are considered. The chemical structure of PHB and PHBV is shown in Figure 1.

### 2.2. Theoretical Background

#### 2.2.1. Gas Transport in Polymeric Membranes

The gas permeability coefficient through a polymeric membrane is defined as the pressure- and thickness-normalised molar flux of gas across a dense polymeric membrane and, as such, is used to characterise the transport of small molecules across the membrane. This process is described using the solution–diffusion model, according to which the permeants dissolve in the membrane material and then diffuse through the membrane down a concentration gradient [38]. Under such assumption, the permeability coefficient $P_{i}$ can be effectively evaluated as a product of solubility coefficient, $S_{i}$, and diffusion coefficient, $D_{i}$.

(1) $$P_{i}=S_{i}D_{i}$$

The separation capability of the membrane is quantified through the selectivity $\alpha_{ij}$, and, in the case of negligible downstream pressure, is equal to the ratio between permeability of gas *i* and gas *j*, which, according to Equation (1), can also be seen as the product of solubility–selectivity, $\alpha_{ij}^{S}$, and diffusivity–selectivity, $\alpha_{ij}^{D}$:

(2) $$\alpha_{ij}=\frac{P_{i}}{P_{j}}=\frac{S_{i}}{S_{j}}\cdot \frac{D_{i}}{D_{j}}=\alpha_{ij}^{S}\cdot \alpha_{ij}^{D}$$

If pure-gas data are used to evaluate the selectivities, then Equation (2) gives the ideal selectivity, which is useful for the first estimate of the separation performance of new materials and its comparison with the existing literature data. However, a more refined design of the process requires an evaluation of the gas–polymer properties under mixed-gas conditions to assess the real multicomponent selectivity of the material.

In the present work, we focused on the solubility–selectivity, which, in the case of the polymers inspected, is the most important contribution to the separation due to the preferential dissolution of CO_2_ in PHAs [7,12]. The sorption coefficient and the solubility–selectivity are estimated under both single- and mixed-gas conditions, as follows:

(3) $$\alpha_{ij}^{S}=\frac{S_{i}}{S_{j}}=\frac{c_{i}/f_{i}}{c_{j}/f_{j}}$$

The fugacity of each gas, $f_{i}$, can be evaluated at different total pressures by using the Peng–Robinson EoS [39]. The binary parameter $k_{CO_{2}/CH_{4}}=0.09$ was used to evaluate the fugacity values in the CO_2_/CH_4_ mixture at all compositions [40].

#### 2.2.2. Molecular Simulations of Gas Solubility

MD simulations can be used to predict the gas solubility in the polymers inspected via a fully atomistic approach, although the procedure is time-consuming. The Widom test particle insertion method [26] can be used to calculate the excess chemical potential of CO_2_ and CH_4_ [18,41] and extract the molar solubility coefficient $S_{i}$, as follows:

(4) $$\frac{1}{S_{i}}=\rho RT\underset{x_{i}\to 0}{\lim}\left[\exp\left(-\frac{\mu_{i}^{ex}}{RT}\right)\right]$$

where ρ is the density of the pure polymer system, and $\mu_{i}^{ex}$ is the excess chemical potential of CO_2_ or CH_4_ at infinite dilution.

The experimental solubility,$\;S_{i}^{sc}$, obtained on semicrystalline samples in previous works [12,25], can be compared to the simulated values, which estimate the solubility coefficient in a hypothetical free amorphous phase, $S_{i}^{am}$, by considering that the sorption is negligible in the crystalline fraction, $X_{C}$, as follows:

(5) $$S_{i}^{am}=\frac{S_{i}^{sc}}{\left(1-X_{C}\right)}$$

#### 2.2.3. Multiscale Procedure for the Parametrisation of the SL EoS and Solubility Calculation

The calculation of gas sorption equilibria in amorphous polymers in the equilibrium region, i.e., at temperatures higher than glass transition temperature $T_{g}$ can be performed by using EoS models. EoS parameters for pure polymers can be obtained from the best fit to PVT data sets. As no experimental data are available in the literature for the polymers studied, the PVT data were obtained in this work in silico, performing MD simulations as described by Minelli et al. [23] and Ricci et al. [24] in previous works, and reported in detail in the following.

In the present work, the SL EoS was found suitable for representing the volumetric properties of the amorphous phase of PHBV copolymers. The EoS exploits a lattice representation of matter to derive thermodynamic relationships for pure components and mixtures. For an accurate description of the model, the reader is addressed to original works by Sanchez and Lacombe [19,42], while the main features are briefly summarised in Section S1 of the Supporting Information.

### 2.3. Simulation and Modelling

#### 2.3.1. MD Simulations—Generation of Initial Configurations

In part 1 of this work, eight different poly(3-hydroxybutyrate-*co*-3-hydroxyvalerate) (PHBV) copolymers were considered with 0, 8, 16, 24, 40, 60, 80, and 100 mol% of 3-hydroxyvalerate (HV) units in order to evaluate the density, solubility parameter, and gas sorption dependence on the molar content of HV units. These will be referred to as PHBV0, PHBV8, …, PHBV100, in the following. The complete description of the initial structure generation and simulation details is given in [25] and is briefly recalled in Section S2 of the Supporting Information.

In the present paper, MD simulations were used to generate PVT data sets for PHBV0, PHBV8, PHB60, and PHBV100, using the same molecular method described in [25] and Section S2, performing the density prediction at three different pressures (0.1, 50, 100 MPa) and ten different temperatures (275, 300, 325, 350, 375, 400, 450, 500, 550, 600 K). Each initial configuration was generated at 600 K and the desired pressure and cooled in a step-wise manner at 50 K/ns. At each intermediate temperature, the system was left to equilibrate for 5 ns in the NPT ensemble (constant number of molecules N, pressure P, and temperature T) before extracting the average density value for the polymer configuration from the final 3 ns of the NPT run. The final density value at each P and T was obtained as an average between three independently generated configurations per polymer. A trial step-wise heating run was performed on the PHBV0 system to check for consistency of the results, returning density values within the standard error of those obtained through the cooling procedure. The PVT data were used to find the thermodynamic relation between state variables in the different polymeric structures and fit them to the SL EoS in order to find the polymer parameters.

The same polymer force field and parameters used to estimate PVT data in this work had been previously used to perform MD simulations of PHBV0, PHBV8, PHBV60, and PHBV100 at 35 °C and post-process the data to extract the CO_2_ and CH_4_ solubility at infinite dilution via Widom insertion method [25]. In that work, the model was validated using different experimental data such as polymer density at room temperature, solubility parameters, and gas solubility data purposively measured. In the present work, we extended the calculation to three more systems, namely, PHBV24, PHBV40, and PHBV80, using the same protocol described in our previous paper and Section S2, with results reported in Figure 5 and Figure S1. The extension was performed to increase the number of available data points for the polymeric systems that would be directly comparable to the predictions made by the multiscale model.

#### 2.3.2. Multiscale Model—Parameterisation of the SL EoS

EoS parameters for pure polymers can be obtained from the best fit of the calculated PVT behaviour to data sets above $T_{g}$. In the present work, the volumetric behaviour of the amorphous phase of PHBV copolymers is obtained from MD simulations, as described by Minelli et al. [23] and Ricci et al. [24], using the protocol reported in Section S2, given no availability of experimental PVT data in the literature for the polymers investigated. Then, the SL EoS was used to represent the volumetric properties of the amorphous phase of PHBV copolymers and the SL EoS was fitted to simulated PVT data to extract the parameters $T^{*}$, $p^{*}$, and $\rho^{*}$. The procedure followed for PHBV copolymers with 0, 8, 60, and 100% of -HV units involved minimisation of the mean absolute percentage error, MAPE, as follows:

(6) $$MAPE=\frac{100}{N_{P}}\cdot \sum_{i=1}^{N_{P}}\left|\frac{y_{i}^{exp}-y_{i}^{calc}}{y_{i}^{exp}}\right|\;$$

where $N_{P}$ is the number of data points considered for regression, while $y_{i}^{exp}$ and $y_{i}^{calc}$ are experimental and predicted values, respectively. The coefficient of determination, $R^{2}$, was also calculated to quantify the accuracy of the prediction.

If the parameters follow a regular trend with the composition of HV monomers, their trend with composition can be represented by considering the copolymers to be a mixture of the homopolymers forming them and using the SL mixing rules with the binary interaction parameter $k_{ij}=0$ to estimate them.

#### 2.3.3. Multiscale Model—Gas Solubility Prediction with SL EoS

The SL EoS was used to calculate pure CO_2_ and CH_4_ solubility in PHBV copolymers, as described in Section S1. The results obtained at 35 °C and 1 bar were compared with experimental data available for PHBV8 and PHBV25 and values obtained from a purely atomistic approach in this paper (for copolymers PHBV24, PHBV40, and PHBV80) and in part 1 of the work (for copolymers PHBV0, PHBV8, PHBV60, PHBV100) [25]. The single-gas solubility in PHBV copolymers obeys Henry’s law, which is reasonable for polymers above $T_{g}$.

The SL model was then used to predict multicomponent gas sorption in the homopolymers PHBV0 and PHBV100 at 35 °C and pressure up to 30 bar for binary CO_2_/CH_4_ mixtures with CO_2_ content of 100, 80, 50, 20, and 0%mol. The binary interaction parameter $k_{ij}=-0.03$ for CO_2_/CH_4_ interactions was used in all SL mixed-gas sorption calculations. This parameter derives from the fitting of CO_2_/CH_4_ mixture experimental PVT data by SL EoS [40,43]. The SL parameters used to describe the behaviour of the two gases in this work are taken from the literature [22] and are reported in Table S2.

## 3. Results and Discussion

### 3.1. MD Simulations

#### 3.1.1. PVT Data

**Figure 2:**
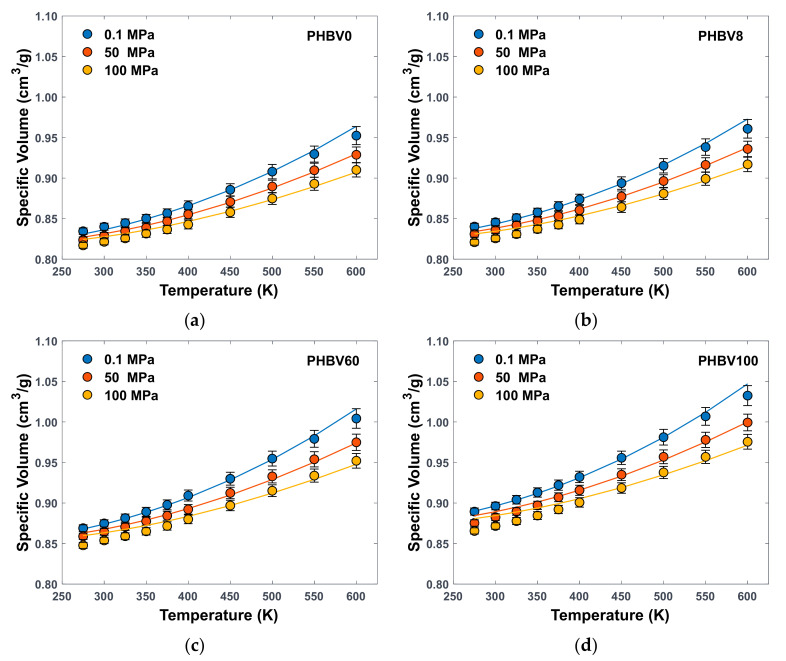
PVT data points obtained from MD for (**a**) PHBV0, (**b**) PHBV8, (**c**) PHBV60, (**d**) PHBV100, fitted by the SL EoS (solid lines).

Figure 2 and Table S1 report the atomistically generated PVT data, together with SL best fitting, for the four investigated structures.

A set of 30 data points was obtained per configuration per polymeric system for a total of 360 values. Each point in Figure 2 is an average value obtained from three different configurations with error bars representing standard deviation. The high-temperature PVT data represent a hypothetical amorphous melt, as thermal degradation of PHBV copolymers generally occurs between ~550 K and 600 K [36,37]. Simulated trends are consistent with the chemical composition, as a higher specific volume is predicted when a higher amount of HV is present in the polymer, in accordance with experimental density values at room temperature [27,44] and the bulkier nature of the ethyl group with respect to the methyl one. Indeed, the presence of HV monomer disrupts the tight packing of the polymer chains. Such effect is directly proportional to temperature, and the simulated thermal expansion coefficient, i.e., the slope of the isobaric curves, is consistently higher in PHBV100 than in PHBV0 at all pressures.

#### 3.1.2. Retrieval of SL Characteristic Parameters

The SL EoS characteristic parameters $T^{*}$, $p^{*}$, and $\rho^{*}$ were obtained by fitting the simulated PVT data estimated in this work for PHBV0, PHBV8, PHBV60, and PHBV100. The fitting of the atomistic PVT data was performed by minimizing the deviation in density prediction between the SL model and the MD-generated data, calculated as MAPE, according to Equation (6). The accuracy of the model fitting can be evaluated by the minimum value of MAPE obtained during the fitting procedure and by the coefficient of determination, $R^{2}$. Both values are reported in Table 1 for all structures. The deviation in density between SL theory and atomistic data, on which the SL model is fitted, is always less than 0.5%. In particular, the maximum error at 25 °C and 1 bar, reported as ${MAPE}_{1bar}^{298K}$ in Table 1, is equal to 0.24% for PHBV0. The value of $R^{2}$ on the full set of data was found to be ≥0.985 in all cases, confirming the high quality of the fit and the good agreement between the molecular model and the macroscopic SL EoS.

**Figure 3:**
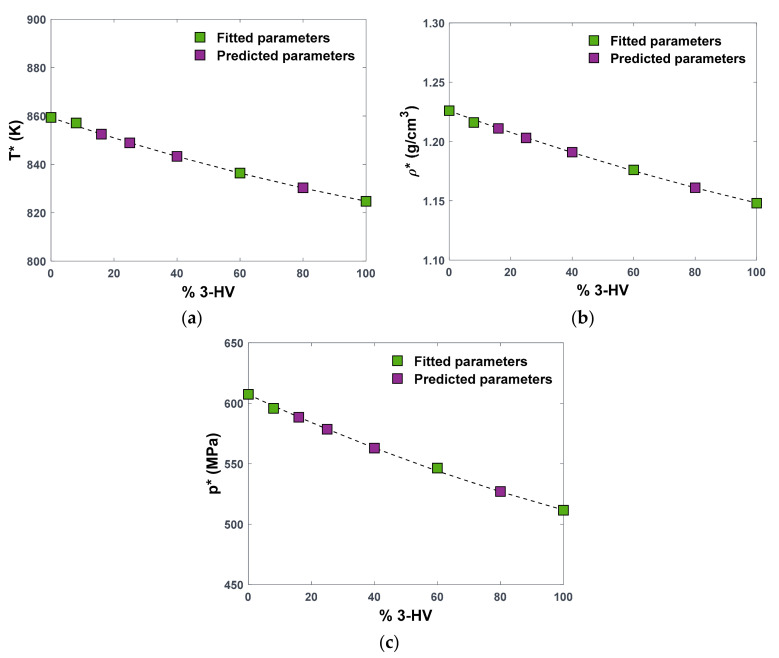
Variation in the characteristic parameters of the SL EoS with HV composition in PHBV copolymers: (**a**) $T^{*}$, (**b**) $\rho^{*}$, and (**c**) $p^{*}$. Parameters were obtained from fitting to MD-generated PVT data (green squares) or predicted considering the copolymer as pseudomixtures using SL mixing rules and $k_{ij}=0$ (dashed lines and purple squares to highlight selected compositions).

**Figure 4:**
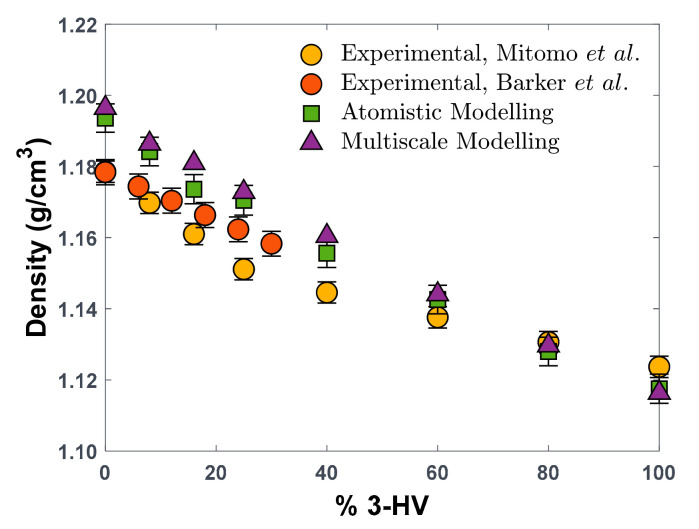
Density of PHBV copolymers at 25 °C as function of the molar concentration of HV units, derived from experimental analysis (yellow and orange circles) [27,44], MD atomistic simulations (green squares) [25], and multiscale method, i.e., SL EoS with parameters estimated via MD-generated PVT data (purple squares).

If plotted against the HV composition of the copolymers, the three SL parameters derived for the four explicitly simulated structures show a clear decreasing linear trend, as can be seen in Figure 3. This is expected, given the trends with chemical structure observed in molecular PVT data for these polymers (Figure 2). As a result, we can assume the copolymers to behave as a mixture of the two homopolymers. Under this assumption, the copolymer behaviour can be described by applying the mixing rules prescribed by SL EoS (Section S1 of the SI), in order to calculate the characteristic parameters of the mixture, i.e., the copolymer, at a desired monomer composition. In this particular case, the binary interaction parameter, $k_{ij}$, which is an adjustable parameter used by the model to evaluate the binary interactions between different species, is set to zero in first approximation, considering also the chemical similarity between the two monomers. As shown in Figure 3, the decreasing trend of $T^{*}$, $p^{*}$, and $\rho^{*}$ with composition in PHBV copolymers is perfectly described by SL mixing rules when $k_{ij}$ is set to 0 (dashed lines), indicating that the interactions between HB and HV monomers can be simply but effectively described by the geometric mean mixing rule. Such a result indicates that the molecular model and the lattice fluid description are consistent in representing the volumetric behaviour of the PHBV polymers and agree in predicting that the copolymers behave as a regular mixture of the two homopolymers. Consistency with experimental density values for the amorphous copolymers, shown in Figure 4, further supports this conclusion.

The main application of this result is the possibility of extending the predictions of polymer behaviour beyond the four structures for which atomistic PVT data were obtained. In particular, given the characteristic parameters of the two homopolymers, PHBV0 and PHBV100, and the desired composition of HV units, the $T^{*}$, $p^{*}$, and $\rho^{*}$ of the specific copolymer are readily obtained by applying the SL mixing rules with $k_{ij}=0$.

As proof of concept, the characteristic parameters for four additional copolymers, PHBV16, PHBV25, PHBV40, and PHBV80, were obtained in this work as described above, and the values are reported in Table 1, indicated by (*) and shown in Figure 3. The indirectly estimated SL EoS parameters were then used to calculate the density at 25 °C and 1 bar for the four polymers, and the calculated values were compared with density values from atomistic simulations. The deviations between density predictions, calculated as ${MAPE}_{1bar}^{298K}$ and reported in Table 1, are in line with values observed for predictions from the direct fitting of the SL model on atomistic data. In the case of PHBV25, the calculated density was compared with the density of PHBV24 obtained from MD simulations in the previous work [25]. Such a result further confirms the validity of the hypothesis that the amorphous part of a PHBV copolymer behaves as a mixture of homopolymers, at least when the volumetric properties are concerned.

Figure 3 shows the dependence of the characteristic parameters on composition. As discussed above, the three parameters decrease with increasing molar concentration of HV monomers, which is consistent with the bulkier nature of the ethyl group present in these monomers. In the lattice–fluid representation, the characteristic pressure of the system, $p^{*}$, is associated with the strength of intermolecular interactions and, thus, with its cohesive energy per unit volume [22], also known as the square of the solubility parameter, $\delta^{2}$, which can be readily estimated for the polymeric system from MD simulations. A decreasing trend of the solubility parameter with composition was observed in the first part of the present work [25], which directly reflects a decreasing trend of $p^{*}$ with the increase in HV monomers. Additionally, $\rho^{*}$ represents close-packed molecular density, reflecting the change in molecular volume due to the change in structure; thus, the decreasing trend with composition is consistent with the induced structural change upon the increase in HV monomers.

Finally, the trend is well represented by SL mixing rules with the binary interaction factor $k_{ij}=0$, which provides an important tool for SL parameter estimation at any desired composition of the PHBV copolymer.

Good agreement between the molecular model and the experimental density data at room temperature, shown in previous work [25], underpins the development of the present multiscale strategy, which yielded a very close agreement with the experimental data on density, reported in Table 1 and Figure 4. This comparison provides the necessary validation for the investigation of gas sorption in PHBV copolymers by using SL EoS characteristic parameters retrieved through the fitting of MD-generated PVT data.

### 3.2. Gas Sorption

#### 3.2.1. Prediction of Pure CO_2_ and CH_4_ Solubility

In part 1 of this work, we reported solubility coefficient values for CO_2_ and CH_4_ in PHBV8 and PHBV25, experimentally measured and appropriately scaled with crystallinity fraction according to Equation (5) [25]. Additionally, values at infinite dilution extracted from the Widom insertion method for PHBV0, PHBV8, PHBV60, and PHBV100 were reported for the two gases and compared with experimental results. In that work, a certain scattering was observed in the simulated solubility data, making it difficult to observe a clear trend of solubility with copolymer composition. For this reason, in this work, we extended the molecular analysis of solubility to three other systems, namely, PHBV24, PHBV40, and PHBV80, in order to achieve a more meaningful basis for comparison between microscopic and macroscopic evaluation of solubility. The results of such analysis can be seen in Figure S1, which shows that the additional solubility data simulated in this paper appear to confirm the non-monotonous trend of solubility with composition. The CO_2_ solubility increases with HV content up to PHBV40 and is 2.5 times higher in PHBV40 than in PHBV8. At molar content of HV higher than 40, a relatively constant solubility value can be observed if the error bar is taken into consideration. This is consistent with experimental trends both from our previous work and the literature [25,45]. On the other hand, a similar trend can be observed for CH_4_, where the solubility value increases with the composition between PHBV8 and PHBV60, then decreases at a higher composition of PHBV100. Finally, the simulated solubility–selectivity seems fairly constant with composition, with higher values of $\alpha^{S}$ observed for PHBV8, PHBV100, and PHBV60, all equal to ~9. In part 1 of this work, such behaviour was attributed to the stronger interactions between CO_2_ and PHBV carbonyl groups, according to the analysis of RDF curves [25].

**Figure 5:**
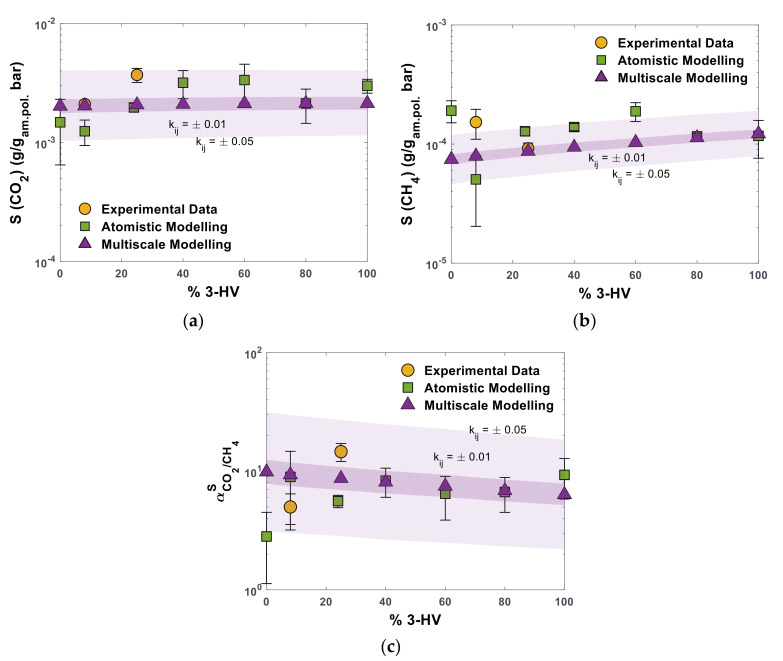
Solubility coefficient values at 35 °C for (**a**) CO_2_, (**b**) CH_4_, and (**c**) CO_2_/CH_4_ solubility–selectivity, on molar basis, in PHBV copolymers as function of the molar concentration of HV units, derived from experimental analysis (yellow circles) [12,25], MD atomistic simulations (green squares) [25], and multiscale method (SL EoS with parameters estimated via MD) (purple triangles). Shaded areas represent the multiscale model predictions using $k_{ij}\neq 0$.

We compared the experimental data and those obtained from atomistic modelling in present and previous works [12,25] with the predictions of the SL EoS. The single-gas solubility values for CO_2_ and CH_4_ are obtained at 35 °C and 1 bar by using SL parameters reported in Table 1 for the polymers and in Table S2 for the two gases. The binary parameter, $k_{ij}$, used to describe gas–polymer interactions, can be treated as an adjustable parameter when the fitting procedure to retrieve SL parameters is performed on solubility data. However, the limited amount of experimental or simulated data or a high uncertainty could lead to misleading results. For this reason, the solubility values for the two gases were predicted assuming $k_{ij}=0$. We estimated the influence of the value of the binary parameter on the solubility prediction by calculating the values of the solubility coefficients using $k_{ij}$ equal to ±0.01 and ±0.05, which are reasonable values for this parameter. This sensitivity analysis is shown in Figure 5 by using differently shaded areas in the plots.

Figure 5a,b shows that the SL EoS, with parameters fitted on MD-generated PVT data, predicts an almost constant value of the CO_2_ solubility coefficient with concentration of HV units, while $S_{CH_{4}}$ increases slightly with composition. This leads to a slight decrease in CO_2_/CH_4_ solubility–selectivity, which changes from ~10 in PHBV0 to ~6.4 in PHBV100, as shown in Figure 5c. Such trends can be theoretically explained by the fact that $p^{*}$ decreases from PHBV0 to PHBV100. As far as the solubility between the polymer and the gas is concerned, similar values of $p^{*}$, that is associated to the solubility parameter δ, are a rough indicator of good compatibility, consistent with the solubility parameter theory. Increasing the HV content brings the copolymer $p^{*}$ closer to the value of CH_4_ (Table S1) and, thus, justifies a higher solubility of this gas in HV-rich polymers. On the other hand, the distance between the $p^{*}$ values of CO_2_ and the copolymers is higher for HV-rich formulations but remains within a limited range throughout the composition range, hence justifying the relatively slight change in CO_2_ solubility observed with composition.

The SL EoS predicts a value of CO_2_ gas solubility 44% lower than the experimental one for PHBV25 with $k_{ij}=0$ [12] and only 3% lower for PHBV8 [25]. The CH_4_ solubility in PHBV25 is predicted by the same method with only a 3.5% negative deviation, while in PHBV8, the underestimation is 47%. If the difference between values derived from molecular and multiscale models is considered, the highest deviation of 64% is observed for CO_2_ in PHBV8 and 61% for CH_4_ in PHBV0. For the selectivity, the deviation is within a factor of 2 from both experimental and simulated values, with the exception of PHBV0, where such a factor reaches a value of 3.5. The SL EoS predictions represent the atomistic and experimental data within their uncertainty if the $k_{ij}$ value is allowed to vary within a range typical for these gas–polymer systems. Such a result is quite promising and non-trivial, given that the ability of an Equation of State to represent volumetric behaviour does not necessarily reflect an equal accuracy in representing the polymer-gas solubility, particularly in the case when no adjustable parameters are used.

Overall, the solubility values predicted by the multiscale model present a smooth monotonous trend with the composition of PHBV copolymers, while atomistic and experimental data present a less clear trend and are characterised by significant scattering. The very low solubility is difficult to determine experimentally with high accuracy, and the uncertainty on the degree of crystallinity, which is used to rescale the experimental data, generates more errors. The accuracy of solubility simulations through the molecular-based Widom insertion method generally decreases as the density of the system increases due to a lower number of successful insertions. Additionally, lower solubility values are intrinsically affected by a higher error, similar to what happens in the experimental case.

The higher regularity of the solubility trend predicted by the multiscale model can be attributed to the fact that the fitting is performed on a larger dataset inherently characterised by lower deviations, specifically, on PVT data obtained from MD simulations, which generally exhibit lower discrepancy compared to solubility values derived from the Widom insertion method [15]. In the present work, the maximum deviation in density values is lower than 1%, while the maximum deviation in solubility values obtained from atomistic modelling is equal to 60% for CH_4_ in PHBV8. Nevertheless, when only a limited amount of experimental data is available for comparison, as in the case of novel materials, the generation of atomistic sorption values is of great importance for extending the predictions of the sorption behaviour to wide ranges of chemical composition.

In view of the solubility and solubility–selectivity results shown in the present section, we believe that PHBV40 and PHBV100 are the most promising candidates in terms of separation capabilities for the CO_2_/CH_4_ gas pair. Indeed, PHBV100 was identified as the most promising candidate in part 1 of this work [25], given its high permselectivity. However, if the degree of crystallinity and mechanical properties are considered, PHBV40 is expected to have the lowest degree of crystallinity among the compositions investigated here [27]. This would promote its ductility and ease of processability when compared to the highly crystalline PHBV0, while its sorption behaviour should be the closest to the values reported here, which refer to a fully amorphous sample.

In conclusion, a general agreement is observed between density values evaluated experimentally at room temperature and via fully predictive MD simulations. The value of gas solubility is inherently affected by larger scattering, both in experiments and in Widom-based simulations, while the multiscale methodology seems to produce more consistent trends at a reasonable computational cost, providing an efficient screening tool for new classes of materials. Additionally, the multiscale method can be used to predict the effect of temperature, pressure, and composition variations in a fast and reliable manner, allowing us to assess the behaviour of the materials in a variety of scenarios, as reported in the following section. Such a result should not discourage conducting fully atomistic investigations of new materials, where the computational effort may be compensated by their entirely predictive character.

#### 3.2.2. Prediction of Solubility of CO_2_ and CH_4_ under Mixed-Gas Conditions

The SL model was used to predict mixture sorption effects in PHBV8 and PHBV25 at 35 °C and at pressures up to 30 bar for binary CO_2_/CH_4_ mixtures with CO_2_ content of 100, 80, 50, 20, and 0%mol. These two polymeric structures were chosen as an example of the practical application of the multiscale model, where the behaviour in mixed-gas conditions can be fully predicted from a combination of experimental and atomistic data.

**Figure 6:**
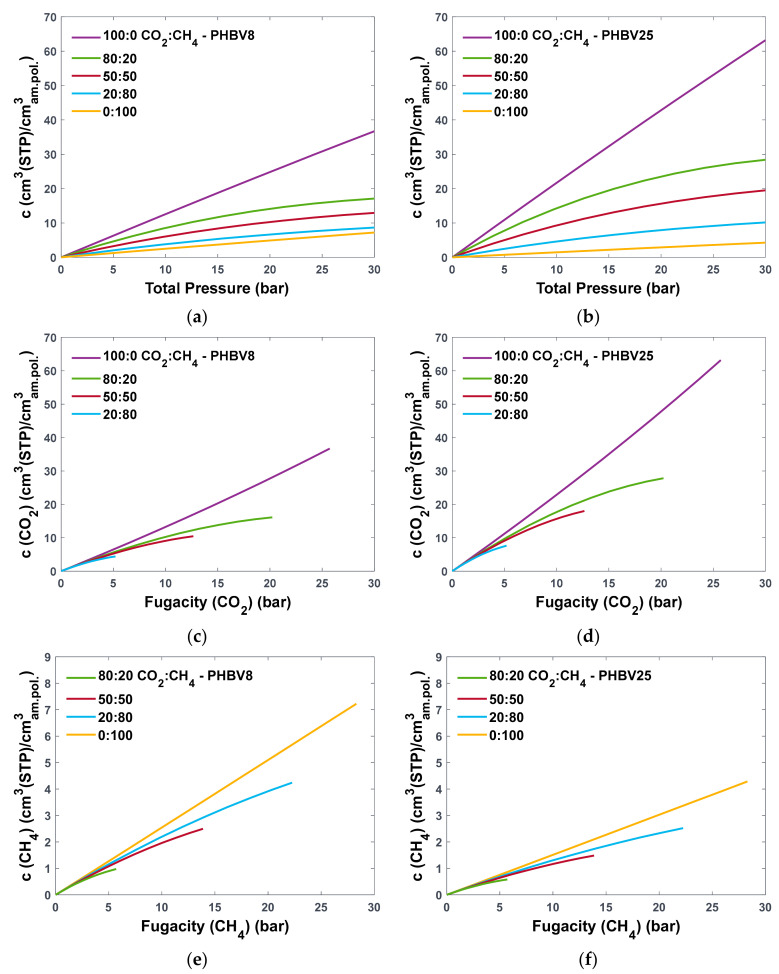
Mixed-gas sorption of binary CO_2_:CH_4_ mixtures with 100, 80, 50, 20, and 0% CO_2_ in (**a**,**c**,**e**) PHBV8 and (**b**,**d**,**f**) PHBV25 at 35 °C, with values of $k_{ij}$ fitted to experimental data. Total concentration of absorbed gases is reported as function of total pressure (**a**,**b**), while CO_2_ (**c**,**d**) and CH_4_ (**e**,**f**) concentrations are reported as function of their fugacity in the mixture.

Figure 6a,b shows the total concentration of absorbed gases at 35 °C in the PHBV8 and PHBV25, respectively, at different total pressures of the mixture. Curves at 100 and 0% of CO_2_ correspond to single-gas sorption isotherms for CO_2_ and CH_4_, respectively. Values of the binary interaction coefficient were obtained from the best fit of pure-gas solubility data, reported in the previous section. The values of $k_{ij}$ equal to −0.002 and −0.043 were obtained for CO_2_ interaction with PHBV8 and PHBV25, respectively, while values equal to −0.069 and −0.005 were obtained for CH_4_ interaction.

The CH_4_ isotherm is linear in both polymers, with the slope equal to the experimental solubility coefficients reported above. The CO_2_ isotherm, on the other hand, is linear at low values of fugacity and only slightly convex to the fugacity axis at higher pressures for PHBV8 and PHBV25. Such behaviour was expected, given the rubbery nature of these polymers at the considered temperature. When mixtures are considered, the linearity is conserved up to ~10 bar of total pressure, while at higher pressures, the total gas concentration drastically deviates from a linear increase and tends towards a plateau. Such an effect is more evident at higher concentrations of CO_2_ in the mixture.

The concentration of each gas can then be analysed separately as a function of the gas fugacity at the investigated pressures, as shown in Figure 6c,e for PHBV8 and Figure 6d,f for PHBV25. As the amount of CO_2_ in the mixture increases, CH_4_ uptake decreases. Similarly, CO_2_ uptake decreases as the amount of CH_4_ in the mixture becomes predominant. Such an effect is more pronounced for CO_2_ and could be attributed to a competitive sorption phenomenon. Indeed, a similar effect for both sorbates of a binary mixture was experimentally observed by Genduso et al. in the case of CO_2_-CH_4_ sorption in a rubbery PDMS [46], and it aligns with the general trend observed for the sorption of several gas mixtures in polymers [13,47].

**Figure 7:**
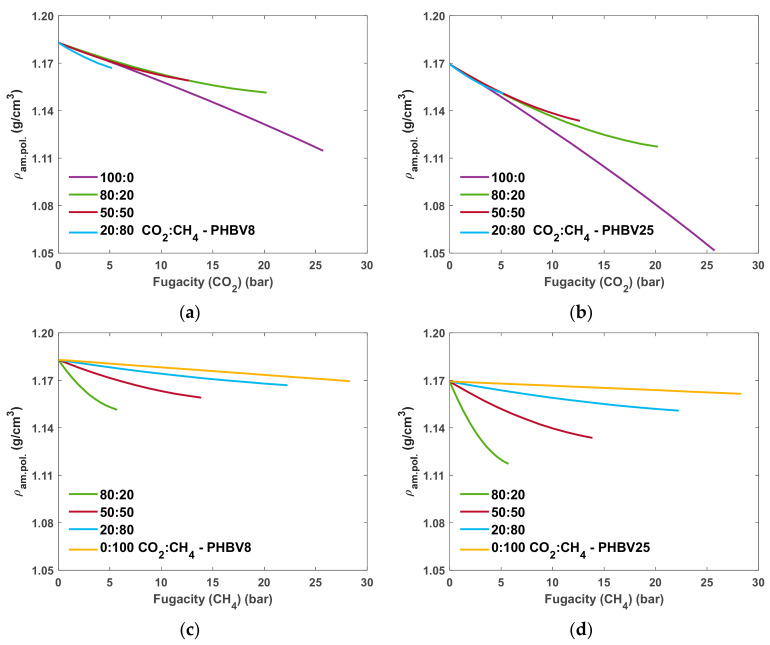
SL-predicted density of the PHBV8 and PHBV25 systems as function of (**a**,**b**) CO_2_ and (**c**,**d**) CH_4_ fugacity in the mixture, obtained with values of $k_{ij}$ fitted to experimental data.

Additionally, the density of the polymer can be studied as a function of gas fugacity in the mixture to better understand the dilation of the matrix during the sorption process. Such analysis is performed for both PHBV8 and PHBV25, and the results are shown in Figure 7. For pure gas sorption, corresponding to 100:0 and 0:100 CO_2_:CH_4_ mixtures, the density change is equal to –5.8% and –10.1% in the case of CO_2_ sorption in PHBV8 and PHBV25, respectively, and to –1.1% and –0.7% in case of CH_4_, at the highest fugacity value of the gas. Thus, both CO_2_ and CH_4_ introduce a certain amount of swelling into polymers upon sorption, with CO_2_ having a more pronounced effect as expected, considering its swelling nature. When CH_4_ is present in the mixture, the swelling effect of CO_2_ is reduced, as shown by the lower absolute change in density of PHBV8 and PHBV25 at concentrations different from 100:0 (Figure 7a,b). When the density is plotted against CH_4_ fugacity (Figure 7c,d), the density of both polymers decreases considerably with increasing amount of CO_2_ in the mixture due to the swelling effect.

Qualitative trends observed for CO_2_ and CH_4_ sorption in PHBV8 and PHBV25 are expected to be followed at all concentrations of HV monomers, given the monotonous behaviour observed in the SL-predicted solubility trends at single-gas conditions. The confirmation of this hypothesis can be seen in Figure S2, where mixed-gas sorption of binary CO_2_:CH_4_ mixtures at different concentrations of CO_2_ is reported for PHBV0 and PHBV100, assuming $k_{ij}=0$ for both gases. Additionally, Figure S3a,b shows the SL-predicted density of PHBV100 as a function of gas fugacity to underline that the trend observed for the polymeric matrix dilation upon gas sorption in PHBV8 and PHBV25 is again conserved at all compositions of these copolymers.

As in the case of pure gas sorption, a sensitivity analysis can be performed to estimate the influence of gas–polymer binary parameter on the value of solubility prediction. This was performed for PHBV100, as an example, calculating the solubility of CO_2_ and CH_4_ using $k_{ij}$ equal to ±0.01 and ±0.05. This analysis is shown in Figure S3c,d by using differently shaded areas in the plots. It should be noticed that, once the appropriate values of $k_{ij}$ are obtained for CO_2_ and CH_4_ through the best fit to single-gas experimental data, the same values should be considered for different compositions of the mixture. Indeed, the sensitivity analysis allows us to evaluate the deviation in gas concentration predictions from the reference case ($k_{ij}=0)$ simultaneously and should not be interpreted as an error bar on gas concentration. The solubility of CO_2_ is only affected by the value of $k_{ij}$ set for CO_2_, while no substantial effect is produced by changing the value of $k_{ij}$ for CH_4_–polymer interaction. When CH_4_ is considered, the value set for $k_{ij}$ of CO_2_–polymer interaction has a slight effect on the sorption capacity of CH_4_ when $k_{{CH}_{4}}=0$, with the highest deviation equal to –1.37% for $k_{CO_{2}}=-0.05$ with 80% of CO_2_ in the mixture. Such effect decreases as the amount of methane in the mixture increases. It is interesting to observe that in the 80:20 CO_2_:CH_4_, the amount of absorbed CO_2_ doubles when $k_{CO_{2}}$ is decreased from 0 to –0.05, which should substantially decrease the sorption capacity of CH_4_ if only the competition effect is present. Thus, it is reasonable to assume that the synergetic effect is also present, where the CO_2_-induced swelling promotes the sorption of both gases, as can be expected for rubbery polymers.

**Figure 8:**
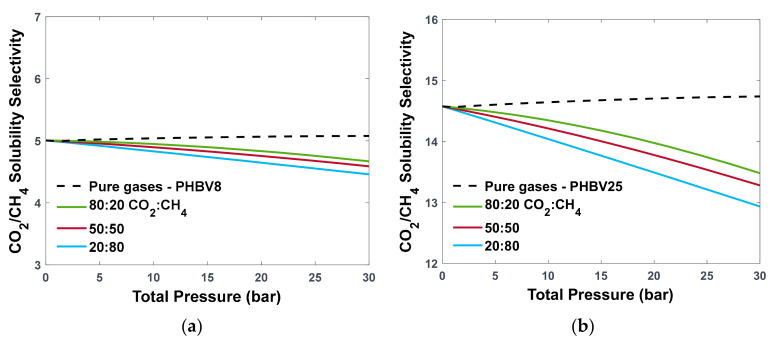
CO_2_/CH_4_ solubility–selectivity predicted by the Multiscale Model for pure- (dashed lines) and mixed-gas (solid lines) sorption in (**a**) PHBV8 and (**b**) PHBV25, with values of $k_{ij}$ fitted to experimental data.

Finally, the solubility–selectivity under mixed conditions was analysed. The decrease in gas sorption in the presence of an additional gas, observed in Figure 6 for PHBV8 and PHBV25, has a direct effect on solubility–selectivity, as shown in Figure 8. Indeed, as opposed to the ideal selectivity that has a slight increase with pressure, the mixed-gas selectivity decreases, and the effect is more significant as the amount of CO_2_ in the mixture decreases. The reduction seems to be more pronounced in PHBV25; however, the solubility–selectivity decreases by 12.2% in both PHBV8 and PHBV100 at 30 bar, the highest investigated pressure, when the CO_2_ concentration reaches 20% in the mixture. Similar behaviour can be observed for different concentrations of HV monomers, as shown in Figure S4 for PHBV0 and PHBV100.

Overall, the SL EoS, fitted on atomistic PVT data, predicts a consistent trend for gas mixture sorption in amorphous rubbery polymers and allows us to estimate the influence of mixture effects on the solubility and solubility–selectivity at the desired operational pressure.

The use of a multiscale modelling method applied for the first time to a biopolymer in the present work, allowed us to expand the gas solubility predictions from four experimental data points, namely, CO_2_ and CH_4_ single-gas solubility in PHBV8 and PHBV25 copolymers, to the whole range of copolymer compositions and operational conditions. Even though these results can be regarded as first-approximation estimates, they can inform and reduce future experimental efforts, allowing us to focus only on the most promising candidates for a specific application.

## 4. Conclusions

The present work extends the knowledge of CO_2_ and CH_4_ solubility behaviour in PHBV copolymers using a hierarchical description of gas–polymer systems. In part 1, we applied MD simulations to study the effect of composition on the separation properties of PHBV copolymers beyond the available experimental knowledge. In the present work, the same atomistic framework was used to produce PVT data for four amorphous polymeric structures in silico, which were fed to a macroscopic SL EoS to retrieve the polymer characteristic parameters. A methodology was provided to retrieve SL parameters for any composition of PHBV copolymers based on the EoS mixing rules. Indeed, the coupling between molecular and macroscopic description allowed us to conclude that the amorphous part of a PHBV copolymer behaved as a mixture of homopolymers, at least when the volumetric properties were concerned.

The CO_2_ and CH_4_ single-gas solubility predictions made by the multiscale model were compared with results obtained in part 1 of the present study, and a satisfactory agreement was found, with a more regular trend in solubility values provided by the thermodynamic description. This confirms the applicability of the multiscale framework to reliably estimate sorption properties in novel materials where an extensive experimental characterisation is not available.

The validation of the SL EoS, fitted on atomistic data, allowed us to extend the evaluation of solubility behaviour to mixed-gas conditions in a computationally cheap fashion at three different compositions of CO_2_:CH_4_ mixture in PHBV0 and PHBV100 polymers. The competitive sorption behaviour, typical of gas sorption in polymers, was observed, with a relatively low reduction in solubility–selectivity in both polymers at the highest investigated pressure of 30 bar.

Overall, parts 1 and 2 of the present work allowed us to fully investigate the gas sorption and transport properties of a specific copolymer series of the PHA family, substantially increasing the information available for this class of biomaterials by bridging molecular and macroscopic description in a multiscale framework.

## Figures and Tables

**Table 1:** SL EoS characteristic parameters for PHBV copolymers retrieved in this work by direct fitting of MD-generated PVT data and indirect estimation using SL mixing rules (denoted by *). Values of MAPE are calculated according to Equation (6).

	PHBV0	PHBV8	PHBV16 *	PHBV25 *	PHBV40 *	PHBV60	PHBV80 *	PHBV100
$T^{*}$ [K]	859.4	857.1	852.5	848.9	843.3	836.4	830.3	824.7
$p^{*}$ [MPa]	607.4	595.8	588.4	578.5	562.9	546.4	527.0	511.5
$\rho^{*}$ [g/cm^3^]	1.226	1.216	1.211	1.203	1.191	1.176	1.161	1.148
$R^{2}$	0.991	0.989	–	–	–	0.988	–	0.985
MAPE	0.330	0.349	–	–	–	0.399	–	0.487
${MAPE}_{1bar}^{298K}$	0.236	0.183	0.626	0.198	0.416	0.126	0.147	0.102

## Data Availability

The data presented in this study are available on request from the corresponding author.

## Supplementary Materials

The following supporting information can be downloaded at https://www.mdpi.com/article/10.3390/polym16192776/s1, Section S1: SL EoS; Section S2: Generation of initial structures and MD simulation details; Figure S1: Solubility coefficient values at 35 °C for (a) CO_2,_ (b) CH_4_, and (c) CO_2_/CH_4_ solubility–selectivity, on molar basis, in PHBV copolymers as function of the molar concentration of HV units, derived from atomistic simulations obtained in this work and the previous one and from experiments; Figure S2: Mixed-gas sorption of binary CO_2_:CH_4_ mixtures with 100, 80, 50, 20, and 0% CO_2_ in (a,c,e) PHBV0 and (b,d,f) PHBV100 at 35 °C, with $k_{ij}=0$. Total concentration of absorbed gases is reported as a function of total pressure (a,b), while CO_2_ (c,d) and CH_4_ (e,f) concentrations are reported as functions of their fugacity in the mixture; Figure S3: SL-predicted density of the PHBV100 system as function of (a) CO_2_ and (b) CH_4_ fugacity in the mixture, calculated assuming $k_{ij}=0$. Gas concentrations in PHBV100 predicted by SL EoS at different values of $k_{ij}$: (c) CO_2_ and (d) CH_4_ concentration; Figure S4: CO_2_/CH_4_ solubility–selectivity predicted by the Multiscale Model for pure- and mixed-gas sorption in (a) PHBV0 and (b) PHBV100, with $k_{ij}=0$; Table S1: Specific volume (in cm^3^/g) obtained from MD for PHBV0, PHBV8, PHBV60, and PHBV100 in the present work at 10 temperatures and 3 pressures; Table S2: SL characteristic parameters used in this work for penetrants, with values retrieved from the literature.
