# Supplementary material for: Modelling Across Multiple Scales to Design Biopolymer Membranes for Sustainable Gas Separations: 2-Multiscale Approach
Source: Polymers (Basel). 2024 Sep 30;16(19):2776. doi: 10.3390/polym16192776 (PMC11478839; doi:10.3390/polym16192776)
Supplement: Supplementary file 1 [file polymers-16-02776-s001.zip › polymers-3207450-supplementary.pdf]

Supporting Information

Modelling across multiple scales to design biopolymer membranes for sustainable gas separations: 2–Multiscale approach

Kseniya Papchenko[1], Eleonora Ricci[1] and Maria Grazia De Angelis[1,2]

[1] Institute for Materials and Processes, School of Engineering, University of Edinburgh, Sanderson Building, Robert Stevenson Road, EH9 3FB, UK; kpapchen@ed.ac.uk (KP), ericci@ed.ac.uk (ER);

[2] Department of Civil, Chemical Environmental and Materials Engineering, DICAM, University of Bologna, Via Terracini 28, 40131 Bologna, Italy;

*Correspondence: Correspondence: grazia.deangelis@ed.ac.uk (MGDA).

Section S1. Sanchez-Lacombe Equation of State (EoS).

The Sanchez-Lacombe EoS [1,2] is obtained by minimising the expression for the Gibbs free energy, derived according to the lattice fluid representation, with respect to volume at constant temperature and pressure. In the Sanchez–Lacombe model, each molecule is described as a flexible chain of r segments immersed in a lattice of cubic cells. Unlike the earlier Flory–Huggins model [3,4], where the lattice is fully occupied and the interactions are repulsive, the Sanchez–Lacombe description allows for the presence of empty cells, that are randomly distributed in the lattice, and assumes attractive pairwise interactions between first neighbours. The interaction between molecule segments and empty cells is assumed to be zero. The total volume is given by the sum of individual lattice cell volumes, required to represent the partially occupied lattice. The Gibbs free energy is then calculated as function of the number of possible configurations of the system, its energy, and its volume, according to statistical mechanics definition.

Within the SL framework, each substance is univocally characterised by three macroscopic parameters, T^*, p^*, and ρ^*. In particular, p^* can be interpreted as a measure of the strength of intermolecular interactions in the substance. The reduced variables, $\tilde{T}$, $\tilde{p}$, and $\tilde{\rho}$ are defined are the ratio between the real system temperature, pressure, and density, and the characteristic SL parameters. The Sanchez–Lacombe EoS allows to evaluate the reduced density, $\tilde{\rho}$, of the system, given $\tilde{T}$ and $\tilde{p}$. The framework is formally identical for pure components and mixtures, provided that the corresponding definition of the reduced variables is used. For the general case of mixtures, one has to know the total number of components in the system, N, the volume fraction of each component, ϕ_i, and the number of lattice sites occupied by a mole of the component i when in mixture, r_i. The number of lattice sites of i in mixture, r_i, is derived from the number of lattice sites occupied by i as a pure component, r_i^0. The value of r_i^0 is calculated from the molar volume of the sites when occupied by i as pure component, v_i^*, according to the definition of $\rho_i^* = M_i/r_i^0 v_i^*$, where M_i is the molar mass of the component i. For polymeric species, the results reach a plateau in the limit of high values of M_i which, for this reason, is often set to an arbitrary high value (10^{30}) in these calculations, if not known experimentally. The molar volume v_i^* is then calculated from a relation analogous to the ideal gas law, written as $p_i^* v_i^* = RT_i^*$. The SL Equation of State can thus be written as follows:

$$\tilde{\rho} = 1 - \exp\left[-\frac{\tilde{\rho}^2}{\tilde{T}} - \frac{\tilde{p}}{\tilde{T}} - \tilde{\rho}\left(1 - \sum_i^N \frac{\phi_i}{r_i}\right)\right] \quad (S1)$$

Equation S1 is implicit in $\tilde{\rho}$, and has to be solved numerically by iteration. The volume fraction of component i is calculated as the weighted average: $\phi_i = \frac{\omega_i}{\rho_i^*} / \sum_N \frac{\omega_i}{\rho_i^*}$, where ω_i is the mass fraction of component i. The volume fraction reduces to unity in case of pure components. The reduced variables

in Equation S1 are defined based on characteristic parameters of the pure component or of the mixture, accordingly.

To obtain the characteristic parameters of the mixture, mixing rules are introduced, defined as follows:

$$\frac{1}{\rho^*} = \sum_i^N \frac{\omega_i}{\rho_i^*} \tag{S2}$$

$$p^* = \sum_i^N \phi_i \cdot p_i^* - \sum_i^{N-1} \sum_{j>i}^N \phi_i \cdot \phi_j \cdot \Delta p_{ij}^* \tag{S3}$$

$$T^* = \frac{p^*}{\sum_i^N \frac{p_i^* \cdot \phi_i}{T_i^*}} \tag{S4}$$

The characteristic binary interactions between species i and j are represented by the value of Δp_{ij}^*, that contains an adjustable binary parameter, k_{ij}, to account for deviations from the geometric mean mixing rule, as follows:

$$\Delta p_{ij}^* = p_i^* + p_j^* - 2(1 - k_{ij})\sqrt{p_i^* \cdot p_j^*} \tag{S5}$$

Once the characteristic parameters of the mixture are obtained, the average molar volume of lattice sites in the mixture, v^*, is calculated as $v^* = RT^*/p^*$. The number of lattice sites occupied by a mole of the component i when in mixture, r_i, can be finally calculated as $r_i = r_i^0 v_i^*/v^*$.

The phase equilibrium condition for the polymer–penetrant mixture is written by equating the chemical potential of the gas in the gaseous phase and in the polymer. For both phases, the chemical potential is calculated as follows:

$$\frac{\mu_i}{RT} = \ln(\tilde{\rho}\phi_i) - \ln(1 - \tilde{\rho}) \cdot \left[r_i^0 + \frac{r_i - r_i^0}{\tilde{\rho}}\right] - r_i - \tilde{\rho}\frac{r_i^0 v_i^*}{RT}\left[p_i^* + \sum_{j=1}^N \phi_i\left(p_j^* - \Delta p_{ij}^*\right)\right] + 1 \tag{S6}$$

To solve the phase equilibrium condition means to calculate the mass fraction of the penetrant in the polymer at the pressures and temperatures of interest. The procedure to do this can be summarised in the following steps:

1. The chemical potential of the pure gas phase, μ_i/RT, is calculated, by solving Equation S1 first and using the obtained value of $\tilde{\rho}$ in Equation S6;
2. The characteristic parameters of the mixture are defined according to Equations S2–S5 as function of the variable ω_i, mass fraction of the penetrant in the polymer;
3. The chemical potential of the gas phase absorbed in the polymer, $\mu_{i,mix}/RT$, is defined as function of the variable ω_i, by coupling the Equation S6 to the Equation S1 for the mixture, given that the gas–polymer density changes with gas mass fraction;
4. The zero of the function $f(\omega_i) = \mu_i/RT - \mu_{i,mix}/RT$ is calculated, while simultaneously solving Equation S1, as defined in the previous step, for $\tilde{\rho}$.

5. The mass concentration of the gas in the polymer is obtained from its mass fraction, as $c_i\left[\frac{g}{g_{am.pol.}}\right] = \omega_i/(1-\omega_i)$.
6. Volumetric gas concentration in the polymer is then obtained as $c_i\left[\frac{cm^3(STP)}{cm^3_{am.pol.}}\right] = c_i\left[\frac{g}{g_{am.pol.}}\right]\cdot\rho_{pol}\cdot\frac{R\cdot T_{STP}}{p_{STP}\cdot MW_i}$, where MW_i is the molar mass of the gas, and $\rho_{pol} = \rho_{mix}\cdot(1-\omega_i)$ under the assumption of $V_{pol} = V_{mix}$.

When a mixture of penetrants at a defined composition is considered, the outlined procedure is followed considering the mass fractions of each penetrant in the polymer as independent variables, with the additional constraint that the sum of all mass fractions calculated in the gas-polymer mixture is equal to unity. Analogously, step 1 has to be modified: the chemical potential of each penetrant in the gaseous mixture is calculated by solving Equation S6 first and using the obtained value of $\tilde{\rho}$ of the mixture in Equation S1 for each penetrant.

MATLAB R2021b software was used to perform both single–gas and mixed–gas SL calculations in the present work, by using in–house scripts. The nonlinear equations and their systems were solved by using numerical solvers implemented in MATLAB software, such as "fsolve".

Section S2. Generation of initial structures and MD simulation details.

Monodispersed polymeric melts at different compositions were generated using the Amorphous Builder plugin of the Materials and Processes Simulations (MAPS) software. All systems were generated at 600 K. After an energy minimization and short equilibration in the NVT and NPT ensemble at 600 K, a temperature ramp of 50 K/ns in the NPT ensemble was applied in order to reach the target value of 298 K. Slower cooling rates were tested as well, and no appreciable difference in results was observed. Each system consisted of 5 chains of 150 monomers each, leading to a molecular weight range between ~13000 ÷ 15000 g/mol and a number of atoms between ~9000 ÷ 11000. For every system, three independent initial configurations were generated and simulated, allowing to extract the average value and standard deviation for each property.

Simulations were performed in full atomistic detail during all equilibration and production runs using the LAMMPS package (3 March 2020 stable release) [5]. The Polymer Consistent force field (PCFF) [6] was used for polymers, CH_4, and polymer–CH_4 systems, while the COMPASS force field [7] was used to describe CO_2 and its interactions with the polymers. All systems were simulated with periodic boundary conditions and the cutoff for Lennard-Jones potential and Coulombic interactions was set to 12 Å. In order to account for the long-range van der Waals interactions, tail corrections were included, while long-range electrostatics were computed with a particle-particle particle-mesh (pppm) method (relative error in forces calculations set to 10^{-6}). Nosé-Hoover thermostat and barostat with dumping

parameters of 10 fs and 500 fs respectively were used for temperature and pressure control. A timestep of 1 fs was adopted for runs in NVT and NPT ensemble.

After the initial configurations were generated and cooled to 298 K, a short equilibration in the NVT ensemble was performed, and production NPT runs of 10 ns were performed at 298 K and 1 bar, in order to extract average values of density and cohesive energy. The systems were then heated to 308 K at 50 K/ns in the NPT ensemble, equilibrated in NVT ensemble, and NPT runs of 10 ns at 308 K and 1 bar were performed. The last 5 ns of the NPT trajectories at 308K and 1 bar were used to perform Widom test particle insertions of CO_2 and CH_4 molecules in 3 polymeric systems, namely PHBV24, PHBV40, and PHBV80. A total of 10^6 insertions per gas per pure polymer system was sufficient for the convergence of the value of the solubility coefficient, according to Equation 4, reported in the main text of the manuscript.

Given the serial nature of Widom calculations, the NPT trajectory of each configuration was split into five trajectories of 1 ns each, and the code was run simultaneously on each split. The running average of the interaction energy between the penetrant and polymer was calculated for each split as function of the number of insertions and compared with the average value of the same property to ensure that the equilibrium is reached. The total trajectory of 5 ns was found to be sufficient for the extraction of solubility values. The repeatability of the results was confirmed by comparing the average values both between the splits of the same trajectory and between different trajectories referred to the same system, although a higher deviation in penetrant solubility prediction was found with respect to polymer density and energy predictions. Under the assumptions made, such deviation should be attributed to the intrinsic uncertainty associated to this methodology rather than the specific system. Finally, the solubility value for each independent box is calculated considering the average and standard deviation of solubility values calculated over five split sections of the trajectory, and the solubility value per system is found considering the average and standard error of three independent boxes.

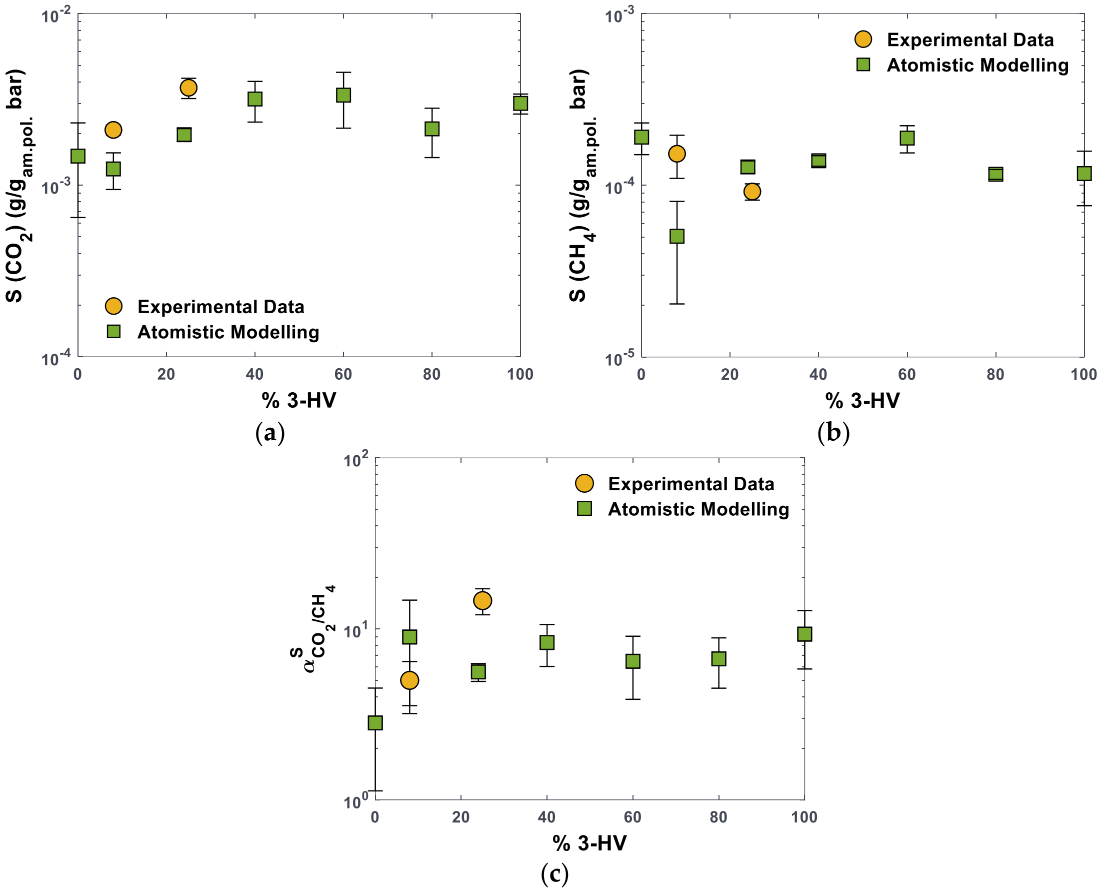

Figure S1. Solubility coefficient values at 35 °C for (**a**) CO_2 and (**b**) CH_4, and (**c**) CO_2/CH_4 solubility selectivity, on molar basis, in PHBV copolymers as function of the molar concentration of HV units, derived from atomistic simulations obtained in this work and in the previous one (green squares) [8], and from experiments (yellow circles) [8,9].

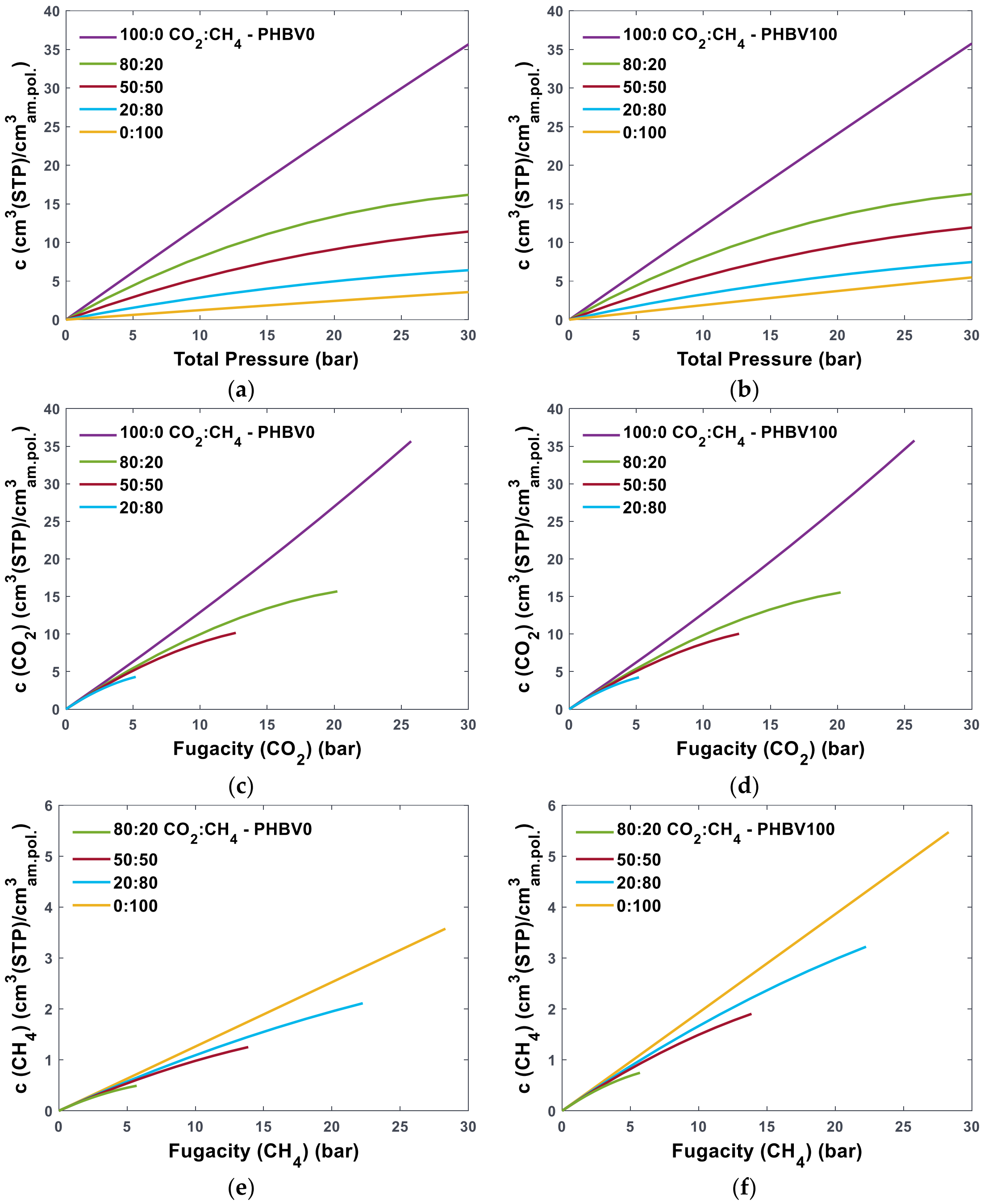

Figure S2. Mixed–gas sorption of binary CO_2:CH_4 mixtures with 100, 80, 50, 20, and 0% CO_2 in (**a**, **c**, **e**) PHBV0 and (**b**, **d**, **f**) PHBV100 at 35 °C, with $k_{ij} = 0$. Total concentration of absorbed gases is reported as function of total pressure (**a**, **b**), while CO_2 (**c**, **d**) and CH_4 (**e**, **f**) concentrations are reported as function of their fugacity in the mixture.

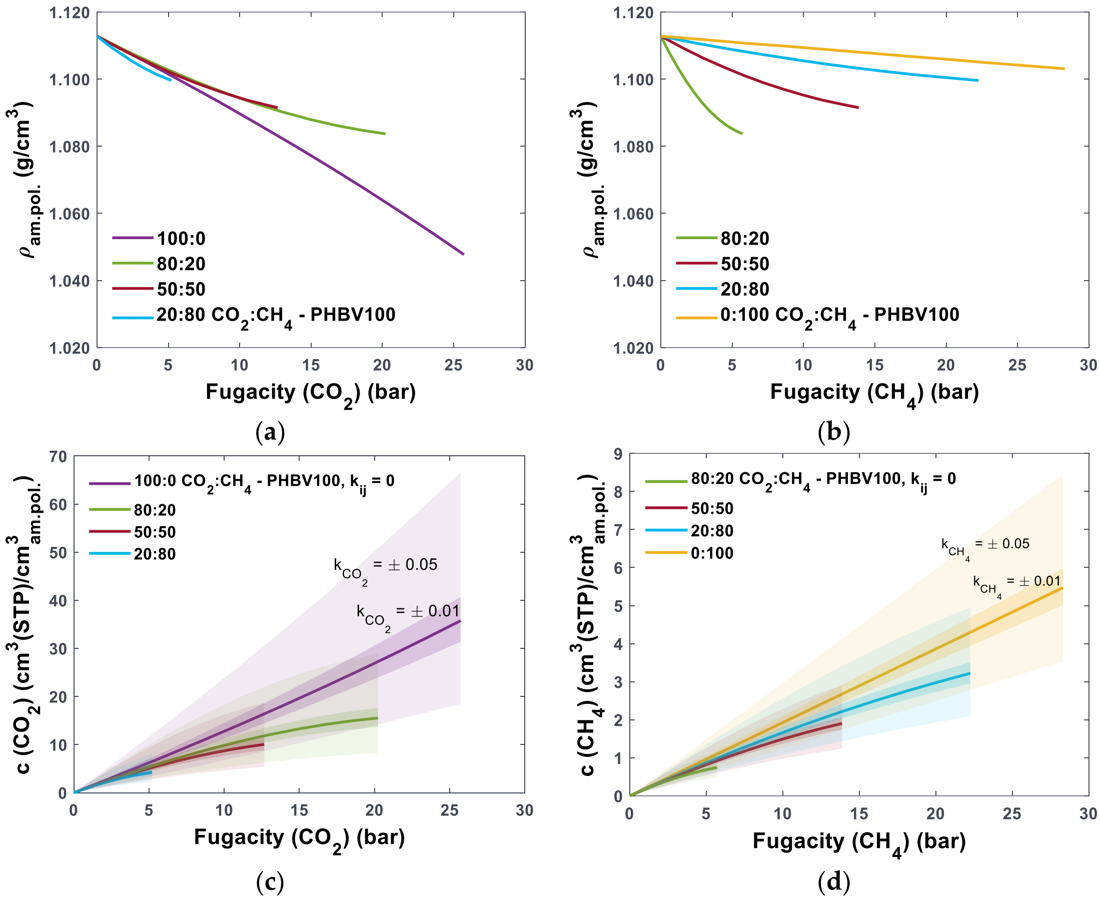

Figure S3. SL–predicted density of the PHBV100 system as function of (**a**) CO_2 and (**b**) CH_4 fugacity in the mixture, calculated assuming $k_{ij} = 0$. Gas concentrations in PHBV100 predicted by SL EoS at different values of k_{ij}: (**c**) CO_2 and (**d**) CH_4 concentration.

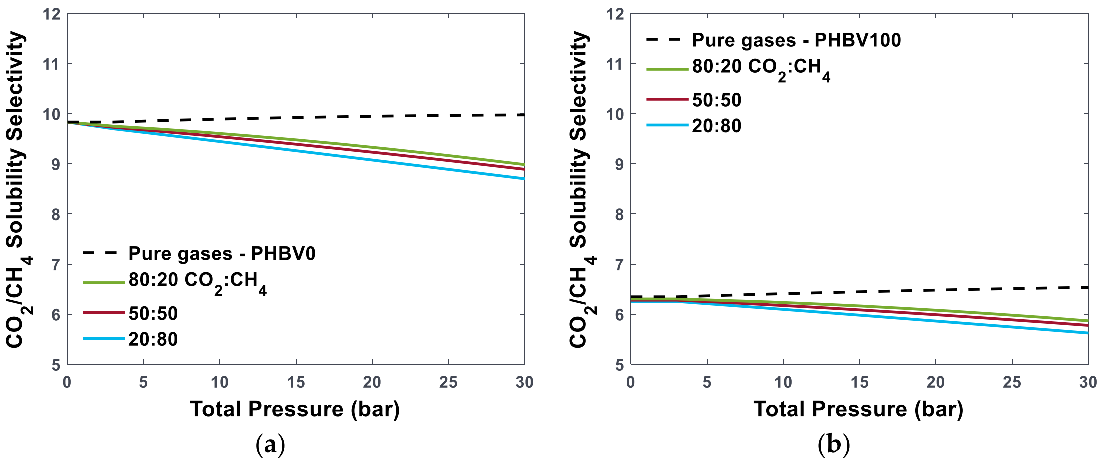

Figure S4. CO_2/CH_4 solubility selectivity predicted by the Multiscale Model for pure- and mixed-gas sorption in (**a**) PHBV0 and (**b**) PHBV100, with $k_{ij} = 0$.

Table S1: Specific volume (in cm^3/g) obtained from MD for PHBV0, PHBV8, PHBV60, and PHBV100 in the present work at 10 temperatures and 3 pressures.

**PHBV0**	0.1 MPa	50 MPa	100 MPa
275 K	0.8344 ± 0.0041	0.8236 ± 0.0038	0.8170 ± 0.0036
300 K	0.8400 ± 0.0044	0.8289 ± 0.0041	0.8214 ± 0.0037
325 K	0.8446 ± 0.0049	0.8344 ± 0.0043	0.8257 ± 0.0041
350 K	0.8501 ± 0.0050	0.8390 ± 0.0048	0.8315 ± 0.0045
375 K	0.8566 ± 0.0055	0.8466 ± 0.0052	0.8366 ± 0.0049
400 K	0.8655 ± 0.0063	0.8553 ± 0.0056	0.8427 ± 0.0054
450 K	0.8856 ± 0.0074	0.8707 ± 0.0068	0.8579 ± 0.0063
500 K	0.9080 ± 0.0089	0.8896 ± 0.0078	0.8748 ± 0.0073
550 K	0.9296 ± 0.0099	0.9097 ± 0.0087	0.8930 ± 0.0081
600 K	0.9524 ± 0.0112	0.9288 ± 0.0096	0.9101 ± 0.0087
**PHBV8**	0.1 MPa	50 MPa	100 MPa
275 K	0.8402 ± 0.0040	0.8309 ± 0.0038	0.8209 ± 0.0036
300 K	0.8456 ± 0.0043	0.8358 ± 0.0040	0.8256 ± 0.0040
325 K	0.8509 ± 0.0049	0.8419 ± 0.0045	0.8308 ± 0.0043
350 K	0.8578 ± 0.0051	0.8472 ± 0.0048	0.8370 ± 0.0046
375 K	0.8654 ± 0.0056	0.8532 ± 0.0052	0.8423 ± 0.0048
400 K	0.8737 ± 0.0063	0.8604 ± 0.0056	0.8488 ± 0.0052
450 K	0.8938 ± 0.0077	0.8774 ± 0.0067	0.8642 ± 0.0066
500 K	0.9151 ± 0.0089	0.8963 ± 0.0078	0.8808 ± 0.0072
550 K	0.9382 ± 0.0102	0.9163 ± 0.0088	0.8991 ± 0.0080
600 K	0.9608 ± 0.0115	0.9360 ± 0.0095	0.9169 ± 0.0089
**PHBV60**	0.1 MPa	50 MPa	100 MPa
275 K	0.8689 ± 0.0042	0.8589 ± 0.0039	0.8477 ± 0.0038
300 K	0.8748 ± 0.0045	0.8644 ± 0.0041	0.8538 ± 0.0040
325 K	0.8814 ± 0.0050	0.8706 ± 0.0045	0.8590 ± 0.0043
350 K	0.8891 ± 0.0056	0.8775 ± 0.0050	0.8650 ± 0.0046

375 K	0.8977 ± 0.0061	0.8843 ± 0.0055	0.8717 ± 0.0051
400 K	0.9092 ± 0.0068	0.8921 ± 0.0059	0.8799 ± 0.0055
450 K	0.9300 ± 0.0080	0.9124 ± 0.0071	0.8969 ± 0.0065
500 K	0.9548 ± 0.0092	0.9329 ± 0.0081	0.9152 ± 0.0073
550 K	0.9791 ± 0.0104	0.9539 ± 0.0094	0.9338 ± 0.0081
600 K	1.0043 ± 0.0121	0.9749 ± 0.0100	0.9521 ± 0.0091
**PHBV100**	0.1 MPa	50 MPa	100 MPa
275 K	0.8894 ± 0.0043	0.8754 ± 0.0040	0.8656 ± 0.0039
300 K	0.8963 ± 0.0046	0.8825 ± 0.0042	0.8716 ± 0.0040
325 K	0.9040 ± 0.0052	0.8895 ± 0.0047	0.8777 ± 0.0044
350 K	0.9130 ± 0.0058	0.8972 ± 0.0051	0.8845 ± 0.0048
375 K	0.9220 ± 0.0063	0.9071 ± 0.0055	0.8920 ± 0.0051
400 K	0.9321 ± 0.0070	0.9157 ± 0.0062	0.9007 ± 0.0058
450 K	0.9558 ± 0.0082	0.9349 ± 0.0072	0.9185 ± 0.0065
500 K	0.9812 ± 0.0096	0.9570 ± 0.0085	0.9377 ± 0.0075
550 K	1.0069 ± 0.0110	0.9779 ± 0.0094	0.9570 ± 0.0081
600 K	1.0325 ± 0.0123	0.9993 ± 0.0103	0.9755 ± 0.0091

Table S2: SL characteristic parameters used in this work for penetrants, with values retrieved from literature [10].

	CO_2	CH_4
T^* [K]	300	215
p^* [MPa]	630	250
ρ^* [g/cm^3]	1.515	0.500

References

[1] R.H. Lacombe, I.C. Sanchez, Statistical thermodynamics of fluid mixtures, J. Phys. Chem. 80 (1976) 2568–2580. https://doi.org/10.1021/j100564a009.

[2] I.C. Sanchez, R.H. Lacombe, Statistical Thermodynamics of Polymer Solutions, 11 (1978) 12.

[3] P.J. Flory, Thermodynamics of High Polymer Solutions, The Journal of Chemical Physics 10 (1942) 51–61. https://doi.org/10.1063/1.1723621.

[4] M.L. Huggins, Some Properties of Solutions of Long-chain Compounds., J. Phys. Chem. 46 (1942) 151–158. https://doi.org/10.1021/j150415a018.

[5] A.P. Thompson, H.M. Aktulga, R. Berger, D.S. Bolintineanu, W.M. Brown, P.S. Crozier, P.J. in 't Veld, A. Kohlmeyer, S.G. Moore, T.D. Nguyen, R. Shan, M.J. Stevens, J. Tranchida, C. Trott, S.J. Plimpton, LAMMPS - a flexible simulation tool for particle-based materials modeling at the atomic, meso, and continuum scales, Computer Physics Communications 271 (2022) 108171. https://doi.org/10.1016/j.cpc.2021.108171.

[6] H. Sun, S.J. Mumby, J.R. Maple, A.T. Hagler, An ab Initio CFF93 All-Atom Force Field for Polycarbonates, J. Am. Chem. Soc. 116 (1994) 2978–2987. https://doi.org/10.1021/ja00086a030.

[7] J. Yang, Y. Ren, A. Tian, H. Sun, COMPASS Force Field for 14 Inorganic Molecules, He, Ne, Ar, Kr, Xe, H_2, O_2, N_2, NO, CO, CO_2, NO_2, CS_2, and SO_2, in Liquid Phases, J. Phys. Chem. B 104 (2000) 4951–4957. https://doi.org/10.1021/jp992913p.

[8] K. Papchenko, E. Ricci, M.G. De Angelis, Modelling across Multiple Scales to Design Biopolymer Membranes for Sustainable Gas Separations: 1—Atomistic Approach, Polymers 15 (2023) 1805. https://doi.org/10.3390/polym15071805.

[9] K. Papchenko, M. Degli Esposti, M. Minelli, P. Fabbri, D. Morselli, M.G. De Angelis, New sustainable routes for gas separation membranes: The properties of poly(hydroxybutyrate-co-hydroxyvalerate) cast from green solvents, Journal of Membrane Science 660 (2022) 120847. https://doi.org/10.1016/j.memsci.2022.120847.

[10] M.G. De Angelis, G.C. Sarti, F. Doghieri, NELF model prediction of the infinite dilution gas solubility in glassy polymers, Journal of Membrane Science 289 (2007) 106–122. https://doi.org/10.1016/j.memsci.2006.11.044.
